# The Promising Effect of Nerve Decompression in Trigeminal Autonomic Cephalalgias: Report of Case Series

**DOI:** 10.3389/fneur.2021.678749

**Published:** 2021-06-07

**Authors:** Mansoureh Togha, Ali Totonchi, Hojjat Molaei, Hossein Ansari

**Affiliations:** ^1^Headache Department, Iranian Center of Neurological Research, Neuroscience Institute, Tehran University of Medical Sciences, Tehran, Iran; ^2^Department of Neurological Surgery, University Hospitals Case Medical Center, Case Western Reserve University, Cleveland, OH, United States; ^3^Sina Hospital, Imam Khomeini Hospital Complex, Medicine School, Tehran University of Medical Sciences, Tehran, Iran; ^4^Department of Neuroscience, University of California, San Diego, San Diego, CA, United States

**Keywords:** paroxysmal hemicranias, chronic paroxysmal hemicranias, hemicrania continua, Trigeminal Autonomic Cephalalgias, pericranial nerve decompression surgery

## Abstract

Trigeminal Autonomic Cephalalgias (TAC) are excruciating headaches with limited treatment options. The chronic forms of TACs, including chronic cluster, chronic paroxysmal hemicrania, and hemicrania continua, are disabling conditions. In addition to drug therapy, there are some studies regarding nerve blocking and nerve stimulation with acceptable results. Here we report four cases of decompression nerve surgery with promising results on pain control in these difficult to treat headaches.

## Introduction

Trigeminal autonomic cephalalgias (TACs) are a group of primary headaches that are characterized by unilaterality of pain and associated ipsilateral cranial autonomic symptoms. Incidence is rare when compared with other primary headache disorders but diagnosis and particularly treatment can prove to be a challenge.

Paroxysmal hemicrania (PHC) and hemicranias continua (HC) are two subtypes of TAC with an absolute response to high daily doses of indomethacin, as one of the diagnostic criteria for the diagnosis based on ICHD-3 (1). Celecoxib has been used as an alternative to indomethacin with partial positive results. There are also anecdotal reports of comparative responses to topiramate, melatonin, and gabapentin (2). Long-term use of indomethacin as the only standard treatment for chronic paroxysmal hemicranias (CPH) and hemicranias continua (HC) could be associated with gastrointestinal complications and potential renal and sometimes adverse cardiac events (3). These potential side effects and complication(s) could sometimes be serious and limit use of indomethacin as a gold standard treatment in the patients. On the other hand, the other drugs mentioned do not usually have enough efficacy to control the pain.

There are a few studies that recommend peripheral nerve block, mainly the supraorbital, supratrochlear, and greater occipital nerves, individually or in combination (4, 5). Nerve blocking is performed on the nerve(s) ipsilateral to pain, which is tender on the exam. It has favorable but transient results. Also, there are a few studies on supraorbital or greater occipital nerve stimulation in the treatment of CPH and HC (6). There are case reports of microvascular decompression surgery in the SUNCT/SUNA subtype of TAC (7). However, to our knowledge, there is no report on nerve decompression on any TAC subtypes including CPH and HC.

We report two cases of hemicrania continua and two cases of chronic paroxysmal hemicrania that have shown impressive persistent response to nerve decompression. This could be a new approach for these severe disabling conditions, particularly for patients who have contraindications or severe adverse events from indomethacin as an only effective treatment in these potentially disabling conditions.

## Case Presentations

- The first case is a 54-year-old woman with a history of continuous headaches for about 9 years. Pain was localized in the right periorbital region extending to the right parietal and occipital area with the severity of about 7 out of 10 on the NRS-11 pain scale. Attacks of excruciating pain associated with ipsilateral tearing and rhinorrhea 3–4 times a day, which has been superimposed on the continuous pain. Her systemic and neurologic exams and brain and orbital MRI were all normal. She had tried different analgesic drugs without remarkable efficacy. With a dramatic response to 225 mg of indomethacin daily, the diagnosis of hemicrania continua was confirmed. Her headache state had fulfilled the diagnosis of hemicrania continua according to the ICHD3 classification (8). Continuous use of indomethacin at that dosage, caused severe gastrointestinal adverse events, which led to stopping it. Celecoxib replaced the indomethacin with only a partial, unsatisfactory response. The addition of other medications, including melatonin, topiramate, lamotrigine, and gabapentin, did not help to alleviate the pain. Multiple and serial peripheral nerve blocks, as opposed to an individual nerve block, using a local anesthetic (combination of lidocaine HCL 2% with bupivacaine HCL 0.5%, 1:1 ratio) performed. These nerves include supratrochlear, supraorbital, and zygomaticotemporal branches of the trigeminal nerve plus greater occipital nerve on right side, which was all tender on exam. The patient experienced substantial pain reduction following each nerve block. However, the transient efficacy and necessity for retreatment led the patient to request a more permanent response, making her eager to volunteer for nerve decompression procedure. She underwent right supraorbital and supratrochlear nerve decompressions through upper eyelid crease incision, and the pressure on the nerves was released over the supraorbital rim by removing the fibrous band, which was kinking the nerves, and removing the adjacent vessels and muscles. Removed tissues were replaced by free fat grafts to cushion the nerve and prevent depression deformity. The right zygomaticotemporal branch of the trigeminal nerve was avulsed endoscopically using two small incisions in the temporal area and dissection at the level of the deep layer of deep temporal fascia; the nerve and accompanying artery were avulsed and cauterized at their emerging point from the deep temporal fascia.

For the right greater occipital nerve, 4 cm midline incision was made at the lower part of the posterior scalp. After the nerve was identified, the trapezius muscle, which was putting pressure over the nerve was removed right over the nerve, and the semispinalis muscle was also removed between the nerve and the midline raphe; then more lateral dissection was carried out all the way to the subcutaneous tissue. On the nerve's path to the subcutaneous tissue, the nerve was released from the pressure and pulsation of the greater occipital artery by removing a segment of artery. Then the subcutaneous fatty tissue flap was prepared to cushion the nerve from further irritation and scarring before closure of the skin. A few days following the procedure, she reported nearly complete freedom from her disabling pain in terms of frequency and severity, similar to the result from the nerve block. In order to confirm that the result is persistent and permanent, we followed up the patient on a regular basis. The efficacy has lasted up to the present, about 3 years after the procedure. The only mild side effect reported by the patient was pain in the operated sites for few days.

- The second case is that of a 35-year-old male with a diagnosis of chronic paroxysmal hemicrania based on ICHD3 classification (8). The patient had a 15-month history of very intense 10–15 min of pain, 5–7 times a day, on his left periorbital and temporal regions associated with ipsilateral conjunctival injection and lacrimation with rhinorrhea. Systemic and neurologic examinations, brain and orbital MRIs, and a paranasal CT scan were all normal. He had a great response to 225 mg of indomethacin per day. The patient reported side effects of indomethacin, including dizziness and heartburn, and therefore we had to stop it. Serial nerve block of the left supraorbital, supratrochlear, and zygomaticotemporal branches of the trigeminal nerve, which all were tender in the exam, was successful in controlling his pain. A combination of lidocaine HCL 2% and bupivacaine Hcl 0.5%, 1:1 ratio used was for nerve block. However, due to the fact that it wore off, we had to repeat the nerve block every 3–4 weeks. The transient effect of the nerve blocks forced him to ask for possible permanent therapies. Gabapentin, melatonin, topiramate, and valproic acid were also tried but did not help. He volunteered for the nerve decompression procedure. The left supraorbital and supratrochlear nerves were decompressed using the same technique previously discussed in the first case above. The zygomaticotemporal branch of the trigeminal nerve was avulsed endoscopically using the same technique as well. After a few days of post-operative pain and congestion at the procedure site, he experienced at least 90% improvement in the frequency of his paroxysmal headaches. We followed up with him over time, and at present, 2 years after the surgery, he is pleased with the nerve decompression surgery treatment as he currently has only occasional mild tolerable pain without the need for any analgesic use. He is also on no preventive medication.
- The third case is a 26-year-old male with a history of continuous pain in his right temporal and periorbital areas with frequent superimposed intense pain associated with conjunctival injection and nasal congestion on the ipsilateral side. His symptoms started about 15 months before our first appointment. His examination was normal except for mild right- sided lid ptosis. Brain MRIs with and without gadolinium were normal. He did not have an acceptable response to various prescribed drugs or botulinum toxin injections prior to referral to our center. He had a dramatic therapeutic response to indomethacin and the impression of hemicrania continua according to ICHD3 classification was accepted for him (8); however, side effects precluded its use for optimal benefit. Nerve blocks of the right zygomaticotemporal and supraorbital branches of the trigeminal nerve (using a combination of lidocaine HCL 2% with bupivacaine Hcl 0.5%, 1:1 ratio) were successful in alleviating his pain temporarily. So he requested to go under nerve decompression of those two selected nerves. After surgery, he reported at least 80% reduction of his continuous pain severity according to his recorded verbal rating scale and also about 80% reduction in intense headache bouts superimposed on his continuous pain, which has persisted for about 11 months post-surgery, and we are continuing our follow-up with him. The only reported transient side effect was pain at the site of operation for few days post-surgery.
- The fourth case is a 22-year-old female with a diagnosis of chronic paroxysmal hemicrania based on ICHD3 criteria (8) with very frequent severe pain for 20–40 min in her right periorbital and temporal region. She experienced these severe headache attacks for about 2 years; however, the diagnosis of chronic paroxysmal hemicrania was delayed for about 1 year before her referral to our center. Her attacks responded well to 175 mg of indomethacin in a day. After about 10 months, she started to complain of gastrointestinal discomfort, and we had to stop indomethacin per a specialist's request. Since her attacks started again, she asked for an alternate treatment. Multiple cranial nerve block on tender nerves was performed for her with good but temporary results. After a few times of nerve blocks, she asked and insisted on a permanent procedure. Regarding our previous successful experience of nerve decompression surgery for a similar disease, this procedure was done for her on the right zygomaticotemporal branch of the trigeminal nerve and on the right greater occipital nerve with the same techniques explained in previous cases. After a few days of recovery of mild pain and burning sensation in the operated site, she reported at least an 80% pain reduction in the frequency of her paroxysmal headaches, and in her follow-up about 6 months after the surgery although she is not completely freefrom pain, she is happy with the relief of about 80% despite being on no medication. Table 1 indicates main characteristics of cases.

**Table 1:** Main information of the cases.

***N***	**Age/Sex**	**Diagnosis**	**Onset of symptoms**	**Brain MRI**	**Medication history**	**Pain region**	**Nerve decompressions localization**	**Side effects of procedure**	**last follow-up after surgery**	**Improvement in pain**
1	54/ F	Hemicrania continua	9 years	Normal	Indomethacin Celecoxib Melatonin, topiramate Lamotrigine Gabapentin	Right periorbital region extending to the right parietal and occipital area	Right supraorbital and supratrochlear nerve Right zygomaticotemporal branch of the trigeminal Right greater occipital nerve	Pain in the operated sites	3 years	Patient reported nearly complete freedom from her disabling pain in terms of frequency and severity
2	35/M	Chronic paroxysmal hemicrania	15 months	Normal	Indomethacin Gabapentin Melatonin, topiramate Valproic Acid	Left periorbital and temporal region	Left supraorbital and supratrochlear nerves	Pain and congestion in the procedure site	2 years	Patient reported at least 90% improvement in the frequency of his paroxysmal headaches
3	26/M	Hemicrania continua	15 months	Normal	Botulinum toxin injections Indomethacin	Right temporal	Right zygomaticotemporal and supraorbital branches of the trigeminal nerve	Pain in the site of operation	11 months	Patient reported at least 80% reduction in his continues pain severity according to his recorded verbal rating scale and also about 80% reduction in intense headache bouts superimposed on his continues pain
4	22/F	Chronic paroxysmal hemicrania based	2 years	Normal	Indomethacin	Right periorbital and temporal region	Right zygomaticotemporal branch of trigeminal nerve and on right greater occipital nerve	Mild pain and burning sensation in the operated site	6 months	Patient reported at least 80% reduction in the frequency of her paroxysmal headaches

## Discussion

Hemicrania Continua is one of the chronic daily headache syndromes classified under the title of trigeminal autonomic cephalgias (TACs) (1). TACs are a group of strictly unilateral headaches with ipsilateral autonomic features. Based on the headache duration, frequency, and medication responsiveness, this group of headaches is classified into different subtypes: hemicrania continua (HC), cluster headaches (CH), paroxysmal hemicrania (PHC), short-lasting unilateral neuralgiform headache attacks with conjunctival injection and tearing (SUNCT), and short-lasting unilateral neuralgiform headache attacks with cranial autonomic symptoms (SUNA).

According to ICHD3, the diagnostic features of HC are strictly unilateral headache lasting more than 3 months, with periods of exacerbation, absolute responsiveness to the therapeutic dose of indomethacin and at least one of the following: (1) autonomic features or (2) restlessness or headache deterioration by movement (8). Most patients remark that the pain is located in the orbital, frontal, and temporal areas. Occipital pain is also reported (9). Episodes of pain exacerbation with ipsilateral autonomic features usually add to the underlying unilateral persistent headache. The headache onset is usually in the third decade of life, but it varies from the first to seventh decades (10). There is a female preponderance, with an estimated female to male ratio of 2:1 (11). Indomethacin is the best treatment in HC, and according to the ICHD-3, the response to indomethacin is necessary to establish the diagnosis. The patient's response to indomethacin is dramatic and fast, but it is important to note that the therapeutic dose of indomethacin is different among patients and ranges from 25 to 225 mg in a day. Though usually the dosage of indomethacin could be reduced several weeks after reaching an effective dose, patients often cannot get rid of the indomethacin usage completely, without headache recurrence. This issue makes the drug's adverse effects more prevalent and the need to find alternative drugs or procedures more important. Like other NSAIDs, renal dysfunction and adverse gastrointestinal effects are the most common hazardous side effects of indomethacin, which cannot be prevented despite protective measures. In these cases that we have outlined, our patients developed side effects from long-term use of indomethacin that were intolerable, and other alternative treatments also failed to control the headaches. Paroxysmal hemicrania is another type of trigeminal autonomic cephalalgias (TACs) with similar features of HC but with pain-free intervals between the strictly unilateral frequent daily headache attacks of 2–30 min duration, associated with ipsilateral autonomic features and restlessness or agitation. In this type of headache, the pain is also responsive to indomethacin. In chronic paroxysmal hemicrania (CPH) headaches continue over 1 year without remission of at least 3 months (8); therefore, as with HC, patients became dependent on indomethacin to control their attacks. As a result, patients are potentially susceptible to side effects from chronic indomethacin use similar to HC. Therefore, finding an alternative effective treatment in patients who cannot use or tolerate indomethacin, always has been a challenge for headache treatment.

With respect to published studies, peripheral nerve block and nerve stimulation, mainly for the supraorbital and greater occipital nerves (3, 6, 7), have been effective in the treatment of paroxysmal hemicrania and hemicrania continua. Previously, it was shown that greater occipital nerve block with steroids significantly improved cluster headache (12, 13). Nerve blocks, however, have transient efficacy, and there are potential complications with nerve stimulators.

Even deep brain stimulation could be an alternative treatment, but it can be associated with complications and intolerability (6). As a first step in treating these four cases, we performed peripheral nerve blocks for our patients, choosing the nerves according to their tenderness in our exam. As a second course of action, we carried out combined nerve blocks as we observed a better effect for these than for individual nerve blocks. When the patient insisted on a permanent method of pain relief and volunteered for the procedure, the nerve decompression procedure was performed with reasonable and long-term results in controlling their headaches.

## Conclusion

We can conclude that nerve decompression procedures could be a safe and mostly efficacious way to treat patients with the incapacitating pain of chronic paroxysmal hemicrania and hemicranias continua in whom medical therapy is not tolerable or sufficient. Planned prospective studies are suggested to find the extent and length of nerve decompression necessary and the possible side effects. However, in view of the low prevalence of these headache types and the fact that the majority of patients can tolerate long-term use of indomethacin, along with the fact that this procedure needs to be done by an experienced surgeon in this field, conducting such a prospective study will be very difficult.

## Data Availability Statement

The original contributions presented in the study are included in the article/supplementary material, further inquiries can be directed to the corresponding author/s.

## Ethics Statement

Ethical review and approval was not required for the study on human participants in accordance with the local legislation and institutional requirements. The patients/participants provided their written informed consent to participate in this study. Written informed consent was obtained from the individual(s) for the publication of any potentially identifiable images or data included in this article.

## Author Contributions

The type of headache diagnosis and patient selection for surgery and writing the manuscript were done by MT. Surgery was performed by AT and HM. HA was coordinator for the surgery and edited the manuscript. All authors approved the final version of the article.

## Conflict of Interest

The authors declare that the research was conducted in the absence of any commercial or financial relationships that could be construed as a potential conflict of interest.
